# Predictive factors of difficulty in lower third molar extraction: A prospective cohort study

**DOI:** 10.4317/medoral.21348

**Published:** 2016-12-06

**Authors:** Joaquín Alvira-González, Rui Figueiredo, Eduard Valmaseda-Castellón, Carmen Quesada-Gómez, Cosme Gay-Escoda

**Affiliations:** 1 DDS, MS. Master degree program in Oral Surgery and Implantology. Faculty of Dentistry – University of Barcelona. Spain; 2 DDS, MS, PhD. Associate professor of Oral Surgery. Professor master degree program in Oral Surgery and Implantology. Faculty of Dentistry - University of Barcelona (Spain). Researcher of the IDIBELL. Barcelona, Spain; 3 DDS, MS, PhD. Professor of Oral Surgery. Professor master degree program in Oral Surgery and Implantology. Faculty of Dentistry – University of Barcelona, Spain Researcher of the IDIBELL. Barcelona, Spain; 4 MD, DDS, MS, PhD. Chairman and professor of Oral and Maxillofacial Surgery, Director of the Master Degree Program in Oral Surgery and Implantology, Faculty of Dentistry, University of Barcelona, Coordinator & Researcher of the “Fundació Institut d’Investigació Biomedica de Bellvitge” (IDIBELL Institute), L’Hospitalet de Llobregat, and Head of Oral and Maxillofacial Surgery Departament, Hospital Quirón Teknon, Barcelona, Spain

## Abstract

**Background:**

Several publications have measured the difficulty of third molar removal, trying to establish the main risk factors, however several important preoperative and intraoperative variables are overlooked.

**Material and Methods:**

A prospective cohort study comprising a total of 130 consecutive lower third molar extractions was performed. The outcome variables used to measure the difficulty of the extraction were operation time and a 100mm visual analogue scale filled by the surgeon at the end of the surgical procedure. The predictors were divided into 4 different groups (demographic, anatomic, radiographic and operative variables). A descriptive, bivariate and multivariate analysis of the data was performed.

**Results:**

Patients’ weight, the presence of bulbous roots, the need to perform crown and root sectioning of the lower third molar and Pell and Gregory 123 classification significantly influenced both outcome variables (*p*< 0.05).

**Conclusions:**

Certain anatomical, radiological and operative variables appear to be important factors in the assessment of surgical difficulty in the extraction of lower third molars.

**Key words:**Third molar, surgical extraction, surgical difficulty.

## Introduction

Third molar removal is one of the most common procedures in Oral Surgery. Several publications have measured the difficulty of this surgical procedure, and most of them tried to establish the main risk factors (1-9). Pedersen Scale classifies third molars based on the Pell & Gregory classification (position of third molar regarding the occlusal plane and the mandibular ramus) and tooth angulation (10). On the other hand, the modified Parant scale uses surgical technique parameters to assess the complexity of the procedure (11,12). Although these classifications have been widely used to determine third molar extraction difficulty, both of them overlook several important preoperative and intraoperative variables. Pedersen scale bases its analysis exclusively on radiological findings, which compromises its sensitivity. Moreover, this classification lacks both intraexaminer and interexaminer reproducibility, which might lead clinicians to an incorrect preoperative evaluation (13,14).

Thus, the present study aims to determine which anatomic, demographic, radiographic and operative factors influence the surgical difficulty of lower third molar removal. Moreover, the authors will develop a predictive model to assess such difficulty.

## Material and Methods

- Study design and sample

A prospective cohort study comprising a total of 130 consecutive lower third molar extractions was performed in the Oral Surgery and Implantology Master degree program of the School of Dentistry (University of Barcelona). The study protocol complied with the guidelines of the Declaration of Helsinki and was approved by the Research Ethics Committee (CEIC) of the Dental Clinic of the University of Barcelona. The study followed the STROBE guidelines for cohort studies (15). All patients signed an informed consent form agreeing to participate in the study. Patients did not receive any financial compensation for their participation in the study.

Inclusion criteria were (1) patient age between 18 and 65 years, and (2) no relevant systemic diseases (American Society of Anaesthesiologists classification ASA I and ASA II). Extractions with forceps and the absence of the adjacent second molar were the main exclusion criteria. The surgical procedure was performed by second or third-year fellows with a similar technique.

The extraction of impacted lower third molars was performed using a similar surgical technique and under local anaesthesia with articaine 4% and epinephrine 1:100.000 (Artinibsa; Inibsa, Lliça de Vall, Spain). The surgical field and all the surgical material were sterile. The surgeon raised a full-thickness flap, which was protected by the Minnesota retractor. Sterile low-speed (20.000 rpm) handpieces and sterile saline solution were used for bone removal and tooth sectioning when necessary. To close the wound, 3-0 silk sutures (Aragó, Barcelona, Spain) were used.

- Study variables: predictors and outcomes

A single researcher (R.F) collected all the study variables. They were divided into 4 groups: demographic, anatomic, radiographic and operative. Demographic variables were age, weight, height and body mass index (BMI). Anatomic variables were facial pattern and lower third molar features (root anatomy and distance between the root and the inferior alveolar canal following the radiological signs described by Rood and Shehab (16). Radiographic variables were descriptors of lower third molar position (Pell and Gregory classification and tooth angulation according to the Winter classification)(10). Finally, operated site (favourable or unfavourable according to the surgeon dominant hand), degree of retention (soft tissue and bone), the need of bone removal, crown sectioning, root sectioning, surgeon’s experience (second or third year fellow), presence of excessive bleeding and the need of verbal or physical support by an assistant professor were classified as operative variables.

Two variables were used to measure the extraction difficulty (outcome variables): operation time (OT; time elapsed between the raising of the full-thickness flap until the complete avulsion of the tooth) and a 100mm visual analogue scale (VAS) filled by the surgeon at the end of the surgical procedure. A VAS of zero suggests the surgery was extremely easy and a VAS of 100 suggests the surgery was extremely difficult.

- Sample size and statistical analysis

The sample size was calculated using the statistical program G * Power 3.0. (Heinrich-Heine-Universität, Düsseldorf, Germany), with an alpha value of 0.05 and a statistical power of 80%.

Data were processed with the Statistical Package for the Social Sciences (SPSS v12.0). Bivariate (t-Student) and multivariate (ANOVA) analysis was computed to measure the association between the predictors and outcome variables. Correlations between patient anatomic variables (age, weight, height and BMI) and outcome variables were also performed. Variables statistically associated with a significant increase (*p*<0.01) of OT and postoperative assessment of surgical difficulty in the VAS were included in a multiple linear regression model. The level of statistical significance was set at *p*<0.05.

Variables statistically associated (*p*<0.05) with a more difficult surgical procedure and in both multiple regression models (VAS and OT) were included in new index of surgical difficult assessment of lower third molar removal proposed by the authors.

## Results

A total of 130 patients with a mean age of 42 years-old (range 16-55 years) and referred for extraction of a lower third molar were enrolled in the study. The average surgical difficulty of the procedures was of 25 mm on the VAS, while the average extraction time was 10.4 minutes. Variables associated with an increased OT and higher difficult surgical assessment are shown in  Table 1.

**Table 1:**
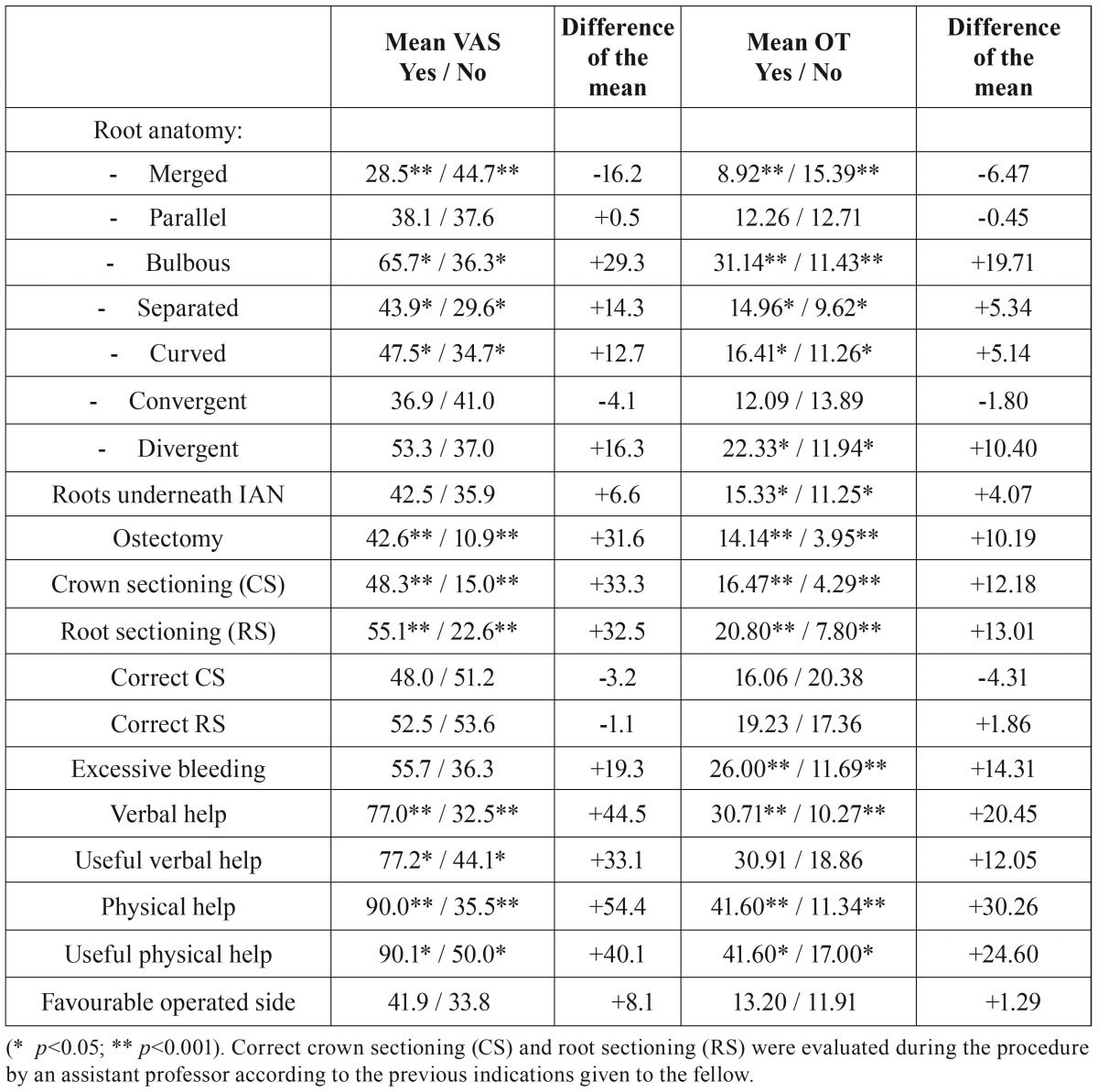
Association between predictive factors and difficulty assessment (VAS) or operation time (OT).

- Operation time (OT)

The average time spent on the procedure was statistically higher in third year residents (16.71min vs. 11.49min; *p*<0.05). Variables such as patient age (*p*<0.05), weight (*p*<0.001) and height (*p*<0.05) showed a positive correlation with OT, whereas BMI did not (*p*>0.05).

Root anatomy of mandibular third molar was closely related with variations in the OT, especially when bulbous or merged roots were involved. Intraoperative variables as the need to perform ostectomy or tooth sectioning (crown and roots) significantly increased the time of the surgical procedure ( Table 1).

Available distal space (Pell and Gregory 123 classification) and a vertical or distal angulation of the third molar (Winter classification) were significantly associated with the OT ( Table 2, Table 3).

**Table 2:**
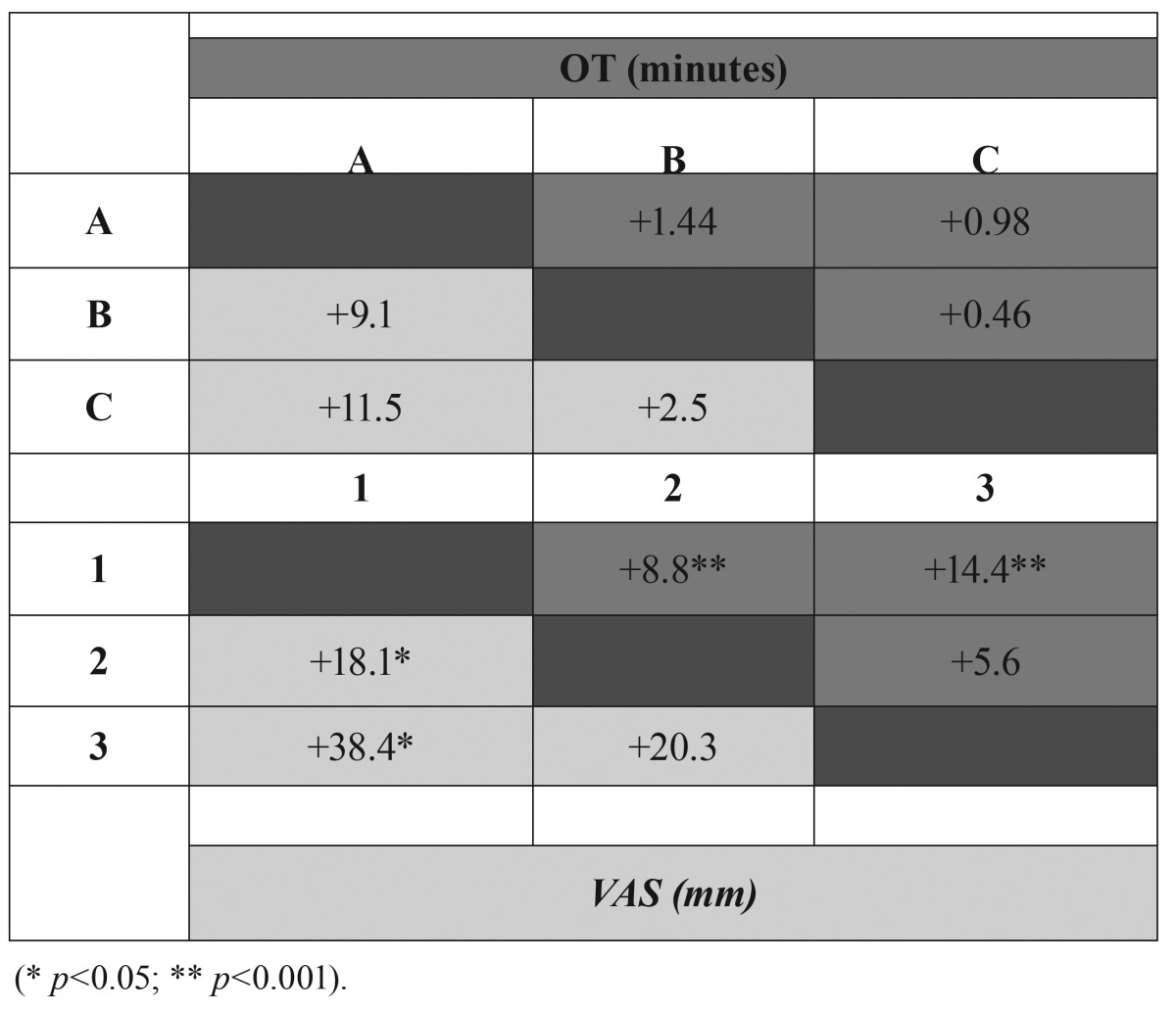
Pell & Gregory (ABC and 123) classification and operation time (OT) and difficulty postoperative assessment (VAS) correlations.

**Table 3:**
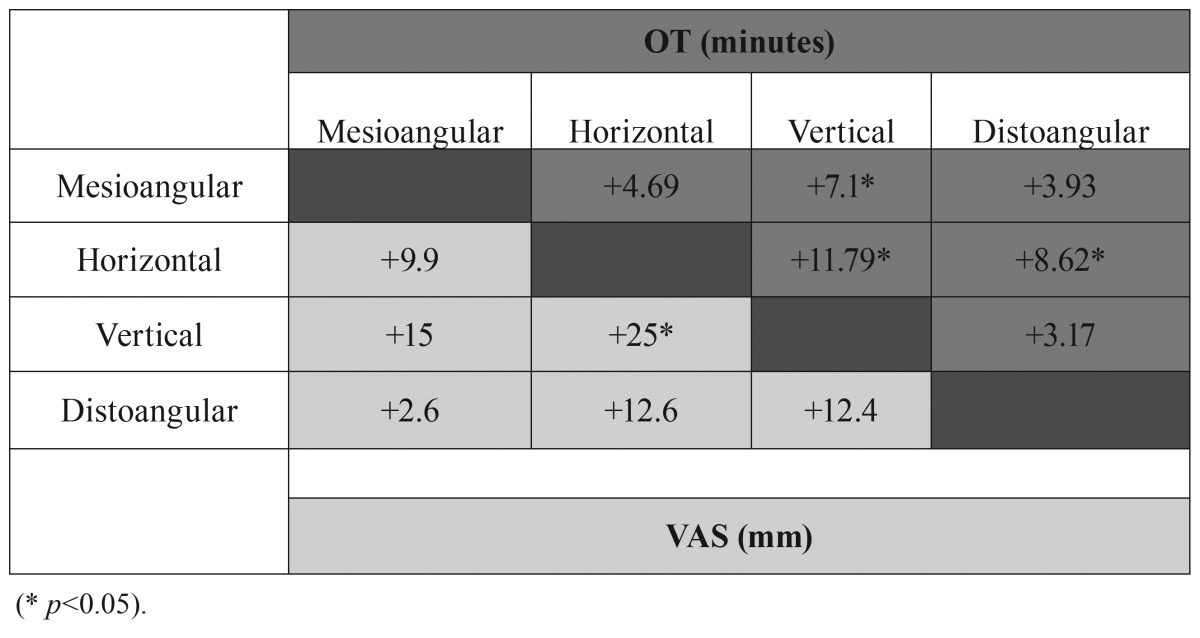
Winter classification and OT and difficulty postoperative assessment (VAS) correlations.

No statistically significant differences were observed between patient’s facial patterns (dolico, meso or brachycephalic) taking into account the time spent in surgery (*p*=0.392). However, the fully erupted molars were extracted significantly faster than teeth with partial retention (*p*=0.017).

- Difficult assessment: Visual Analog Scale (VAS)

The average difficulty assessment of the surgery was higher in third year residents (48.3mm vs 34.9mm; *p*<0.05). Factors such as age (*p*<0.05), weight (*p*<0.001) and BMI (*p*<0.05) showed a positive correlation with VAS scores, whereas height did not (*p*>0.05).

Surgeons considered the extraction of third molars with merged roots as an easy procedure ( Table 1). On the other hand, when bulbous roots were present, high VAS values were recorded. The degree of retention of the third molar as well as the need to perform ostectomy or tooth sectioning significantly increased the surgical difficulty (VAS). Interventions with partial or complete bone retention were considered more difficult when compared with erupted third molars (*p*= 0.036 and *p*=0.040). In contrast, no statistically significant differences were seen regarding the patient’s facial pattern (dolico, meso or brachycephalic) (*p*=0.333).

Angulation of the third molar didn’t seem to change the surgeons’ perception of the operation difficulty, whereas third molars close to the mandibular ramus (Pell and Gregory 123 classification) were considered as more difficult ( Table 2, Table 3).

- Multiple lineal regression models: OT and difficult assessment on VAS

The multiple regression model, taking into account OT as a dependent variable, included the following independent variables: crown sectioning, root sectioning, weight, bulbous roots, divergent roots, Pell and Gregory 123 classification and degree of retention. All variables showed a statistically increased operation time when present or performed (*p*< 0.05) (R2=0.57, regression contrast: *p*=0.011).

Considering VAS (as dependent variable), the multiple regression model included the following independent variables related to surgery: crown sectioning, root sectioning, weight, bulbous roots, Pell and Gregory 123 classification and operated site. All variables were associated to a more difficult surgery (*p*< 0.05) (R2=0.52, regression contrast: *p*=0.041).

Weight, distal space available for eruption (Pell & Gregory 123 classification), bulbous roots and the need to perform crown and root sectioning, were factors statistically associated with both outcome variables (OT and VAS) for surgical difficult assessment. Thus, a classification of the surgical difficulty of this procedure is proposed by the authors and presented in  Table 4 taking into account patients’ general features (weight), radiological and anatomical characteristics of the third molar (distal space and root anatomy), and surgical technique details (crown and root sectioning). Weight values were analysed in a ROC curve considering both outcome variables and separated according to the gender.

**Table 4:**
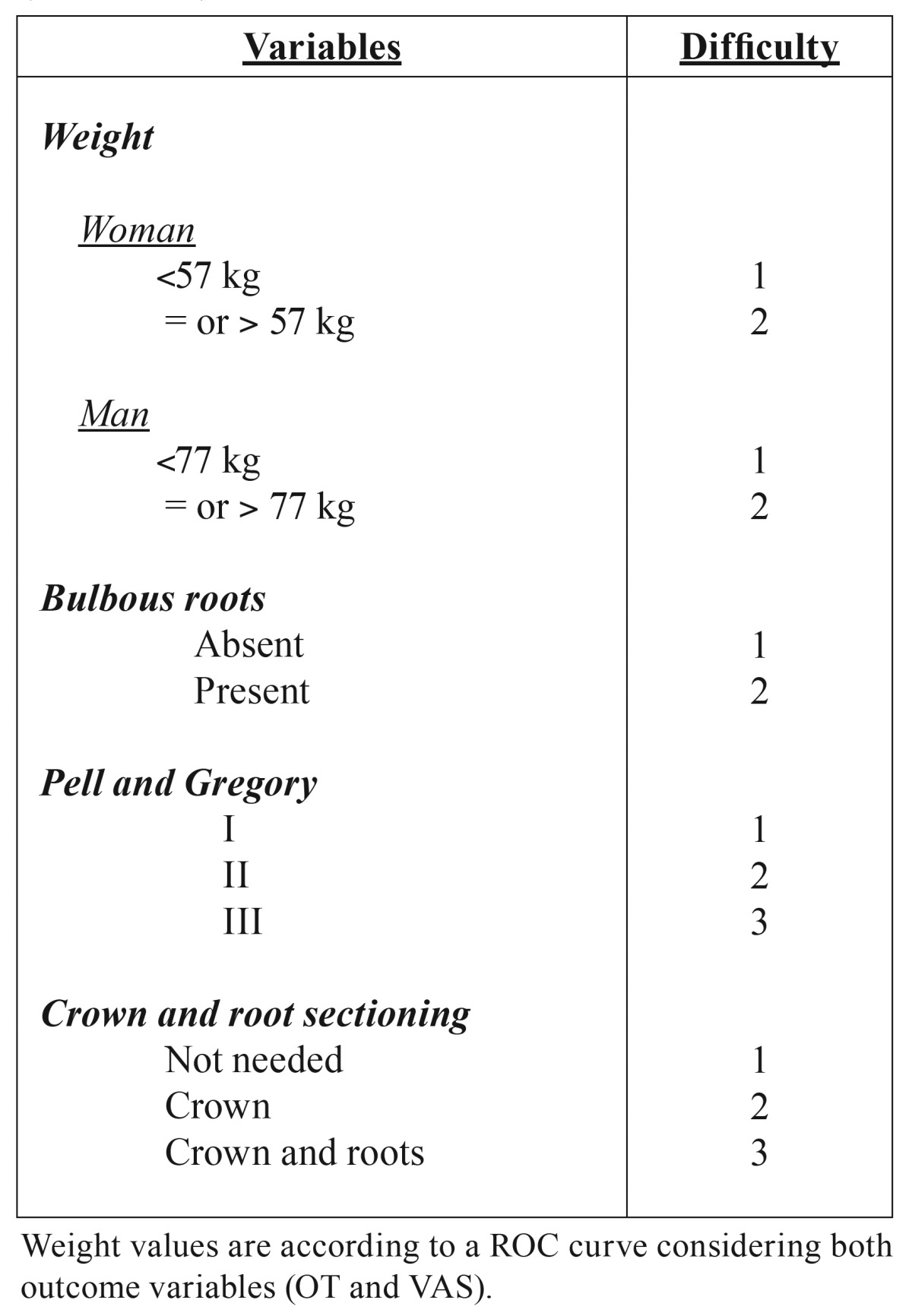
Assessment of difficulty regarding anatomic, radiographic and operative variables. Surgical procedure (Range 4 -10): easy (from 4 to 5), moderate (from 6 to 8) and difficult (from 9 to 10).

## Discussion

Third molar extraction difficulty assessment has been traditionally based on radiological findings and specific anatomic features (Pedersen scale) (10). However, important variables are often overlooked. The present study, supports this statement since patients’ individual factors like weight, radiological characteristics (Pell and Gregory classification), anatomical variables like bulbous roots and the need for bone removal or tooth sectioning have been included in both lineal regression models.

Visual Analogue Scale (VAS) has been previously used to evaluate the complexity of surgical procedures. Likewise, operation time (OT) seems to be an adequate measure of difficulty due to its objectivity and clinical relevance (12,17-20). Furthermore, both variables allow comparisons.

According to a report by Garcia *et al.* (21), Pell and Gregory classification, widely employed by dentists to measure third molar removal difficult, does not seem to be a reliable predictor of surgical difficulty. Our results also seem to support this statement, since the depth of impaction (classes A, B and C), did not show a significant association with the postoperative VAS score nor with the OT. Another important limitation of Pell and Gregory classification is the lack of intra- and inter-examiner reproductibility (12-14,22). Taking into consideration all these factors, radiographic factors alone seem to be insufficient to predict lower third molar extraction difficulty.

Prolonged duration of a surgery is associated not only to a more complex procedure but also poorer postoperative period. The selection of the surgical technique used in the extraction of third molars plays a crucial role in the postoperative period. In our study the need for bone removal and crown and/or roots section were associated with increased postoperative time. Mavrodi *et al.* (23) in their study show that using an elevator on the buccal surface of the tooth seems to be a reliable method to extract impacted third molars safely, easily, quickly and with the minimum trauma to the surrounding tissues. Besides, Gay-Escoda *et al.* (24) suggest that partial closure of the flap without suturing the relieving incision after surgical extraction of lower third molars reduces operating time and it does not produce any postoperative complications compared with complete closure of the wound.

Age was significantly correlated with longer OT and higher VAS scores. One possible explanation to this result might be related with the increased bone density in older patients, and to a certain degree of ankylosis (5,9,25). Patients’ weight was also linked to more difficult surgical procedures, probably because of the reduced visibility of the surgical area in overweight patients. A report by Susarla *et al.* (2) also showed that this variable leads surgeons to an inadequate assessment of the surgical difficulty. On the other hand, differences observed in both outcome variables (VAS and the OT) between second and third year fellows were probably due to the learning curve and individual dexterity of the surgeon in the context of a Master’s degree programme in oral surgery and implantology. Besides, the need for help by an assistant professor (independent of whether verbal or physical) not only meant more time to perform the procedure but also it was considered as more difficult than expected.

Regarding root anatomy, bulbous roots were associated with longer OT and higher VAS scores whereas, merged roots made extractions easier. Third molars in close relation with the inferior alveolar nerve lead to longer surgical procedures. In these situations, surgeons take special care in order to avoid nerve injuries, and therefore the OT might be extended. This fact is also supported by Benediktsdóttir *et al.* (17), in which the authors found that a radiographic relation between the third molar and the mandibular canal doubles the risk for an extended OT.

One important feature that significantly influences the surgical difficulty is the presence of an atypical root anatomy (4). Although the majority of these alterations can be assessed preoperatively in panoramic radiographs, some of them may go unnoticed by the professional and can lead to an underestimation of the extraction difficulty. A possible solution to this problem is the use of Cone Beam Computed Tomography (CBCT) images, which is gaining much acceptance in this type of intervention. Likewise, excessive bleeding, which also increases the complexity of the procedure, is a variable that cannot be predicted. These factors explain why lower third molar extraction difficulty is so complex to predict, since surgeons must assess a huge number of variables, some of which are not available before surgery.

The interpretation of the results should be made with caution for several reasons. First, the study was performed in a University training program. Thus, the degree of postoperative surgical difficulty registered by a fellow may not be representative when compared to an experienced professional.

On the other hand, patient’s anxiety degree should always be considered before surgery provided that the procedure is performed under local anesthesia. Although the extraction of a third molar is a common and well known intervention in oral surgery, degree of preoperative anxiety was associated with more difficult interventions as Aznar *et al.* (26) concluded in their study. These authors stated that extended operation time and higher rates of surgical difficulty were associated with higher levels of patients anxiety, among other variables that are in common with the results found in our study. In the same way, Balaguer-Martí *et al.* (27) indicated in their study that patient satisfaction directly depends on the efficiency of the surgeon and clarity of the clinical information received about the procedure, being both predictive factors that may help clinicians to provide optimal care for impacted third molar surgery patients.

Despite the limitations, the data presented in this study confirm the findings of other authors in previous reports and highlights the importance of certain variables in the surgical difficulty assessment of the third molar extraction (1-7,9,17,28-30).

## Conclusions

According to the present report, weight, distal space available for eruption (Pell & Gregory 123 classification), bulbous roots and the need to perform crown and root sectioning are factors associated to a more complex procedure and are variables that should always be included in the patient’s preoperative evaluation. Other variables like the distance between the inferior alveolar nerve and the third molar, excessive bleeding or atypical root anatomy also seem to significantly influence the surgical difficult.
